# Prostate Cancer Mortality-To-Incidence Ratios Are Associated with Cancer Care Disparities in 35 Countries

**DOI:** 10.1038/srep40003

**Published:** 2017-01-04

**Authors:** Sung-Lang Chen, Shao-Chuan Wang, Cheng-Ju Ho, Yu-Lin Kao, Tzuo-Yi Hsieh, Wen-Jung Chen, Chih-Jung Chen, Pei-Ru Wu, Jiunn-Liang Ko, Huei Lee, Wen-Wei Sung

**Affiliations:** 1 Department of Urology, Chung Shan Medical University Hospital, Taichung, Taiwan; 2 School of Medicine, Chung Shan Medical University, Taichung, Taiwan; 3 Institute of Medicine, Chung Shan Medical University, Taichung, Taiwan; 4 Department of Medical Education, Chung Shan Medical University Hospital, Taichung, Taiwan; 5 Department of Surgical Pathology, Changhua Christian Hospital, Changhua, Taiwan; 6 Department of Medical Technology, Jen-Teh Junior College of Medicine, Nursing and Management, Miaoli, Taiwan; 7 Graduate Institute of Cancer Biology and Drug Discovery, Taipei Medical University, Taiwan.

## Abstract

The variation in mortality-to-incidence ratios (MIRs) among countries reflects the clinical outcomes and the available interventions for colorectal cancer treatments. The association between MIR of prostate cancer and cancer care disparities among countries is an interesting issue that is rarely investigated. For the present study, cancer incidence and mortality rates were obtained from the GLOBOCAN 2012 database. The rankings and total expenditures on health of various countries were obtained from the World Health Organization (WHO). The association between variables was analyzed by linear regression analyses. In this study, we estimated the role of MIRs from 35 countries that had a prostate cancer incidence greater than 5,000 cases per year. As expected, high prostate cancer incidence and mortality rates were observed in more developed regions, such as Europe and the Americas. However, the MIRs were 2.5 times higher in the less developed regions. Regarding the association between MIR and cancer care disparities, countries with good WHO ranking and high total expenditures on health/gross domestic product (GDP) were significant correlated with low MIR. The MIR variation for prostate cancer correlates with cancer care disparities among countries further support the role of cancer care disparities in clinical outcome.

Prostate cancer is a common malignancy characterized by a generally slower progression than other cancers and is a major cause of cancer deaths in men^1^,^2^,^3^. Moreover, gradual increases in prostate cancer incidence have been reported^4^. This increased detection is most likely attributable to cancer screenings that measure prostate-specific antigen (PSA) levels^1^,^5^. There were 1.4 million cases of prostate cancer and 293,000 deaths worldwide in 2013^2^. However, the incidence varied among countries because of differences in the coverage of PSA screenings; the highest number of screenings occurred in Western countries^4^,^6^,^7^. In contrast, the incidence of prostate cancer is much lower in Asia and Africa^8^,^9^. Furthermore, the geographic distribution of prostate cancer incidence in Europe shows that the probability of being diagnosed with prostate cancer is closely associated with prior migrations and settlement histories^10^. Furthermore, prostate cancer is thought to have a strong ethnic propensity, and there is a higher prevalence among Europeans and African Americans^10^.

With regard to mortality rates, prostate cancer is a common cause of cancer death in Western countries^1^,^2^,^3^. The age-standardized, 5-year relative survival of patients in Europe was approximately 83.4%, whereas for those under 80 years of age in the United States, this rate was more than 97%^11^. Current prostate cancer treatments have shown a trend toward personalizing treatments. Future treatment approaches may be guided by cancer DNA sequencing, and personalized drugs could target the weaknesses of certain cancers^12^. All of these improvements suggest that the health care system may be able to improve cancer screenings and treatments of prostate cancer. Therefore, in countries with better health care systems, the mortality-to-incidence (MIR) ratio should be low.

The purpose of this study was to clarify the association between different factors, including human development, World Health Organization (WHO) ranking, region, total expenditure on health/gross domestic product (GDP; e/GDP), life expectancy, and crude rates of incidence and mortality for prostate cancer. Our results could provide a comprehensive overview of the relationship between MIR and health disparities for various countries.

## Materials and Methods

Cancer epidemiological data were obtained from the GLOBOCAN 2012 database, a public database that provides contemporary estimates of the incidence of cancer mortality and prevalence of major cancer types for 184 countries worldwide. The GLOBOCAN 2012 database is maintained by the International Agency for Research on Cancer. The detailed summarized data from GLOBOCAN 2012 were found in an article by Torre LA and colleagues^3^. The inclusion criterion for the selected countries in this investigation of prostate cancer was an incident case number larger than 5,000 diagnoses; 35 countries were selected. The WHO ranking was obtained from the WHO’s Ranking of the World’s Health Systems, which is maintained by the WHO. The e/GDP and life expectancy of 2012 were obtained from the World Health Statistics 2015, which is the annual compilation of health-related data for its 194 member states.

The MIR was defined as the ratio of the crude rate of mortalities and the crude rate of incidences^13^; in this study, the percentage of the total number of mortalities and the total number of incidences was used. The associations between the MIR and other factors among various countries were estimated by simple linear regressions. R-squared changes and analysis of variance (ANOVA) were determined using SPSS statistical software version 15.0 (SPSS, Inc., Chicago, IL). *P* values < 0.05 were considered statistically significant. A scatterplot was generated using Microsoft Excel 2010.

## Results

### The incidence and mortality rates of prostate cancer were higher in more developed regions than in less developed regions

To understand the global trend of prostate cancer, we analyzed the incidence and mortality rates according to region. The results are summarized in Table 1. Overall, the crude incidence and mortality rates of prostate cancer were 30.8 and 8.6, respectively. Both the incidence and mortality crude rates were higher in more developed regions than in less developed regions (incidence: 122.4 vs. 12.0; mortality: 23.4 vs. 5.6, respectively). Regarding human development levels, the regions with high human development levels had higher crude rates of incidence and mortality of prostate cancer than those with low human development levels (incidence: from 129.0 to 7.3; mortality: from 23.1 to 6.0, respectively). With regard to the WHO regions and continents, the WHO Europe region and Americas region had much higher crude rates of incidence and mortality compared with other regions. With respect to continents, North America had the highest incidence rate, but the highest mortality rate was reported in Europe.

### The mortality-to-incidence ratios for prostate cancer are high in less developed regions

As we know, the MIR demonstrates the related outcome of patients with a certain disease, and we investigated the MIR according to region. The world MIR for prostate cancer was 28.1%. There were higher MIRs of prostate cancer in the WHO Africa region, the East Mediterranean region, and the Southeast Asia region (72.5%, 65.3%, and 64.7%, respectively). With regard to continent, Africa had the highest MIR compared with other regions (71.9%).

### World Health Organization ranking and total expenditure on health/GDP were significantly associated with the mortality-to-incidence ratios for prostate cancer

**Figure 1:**
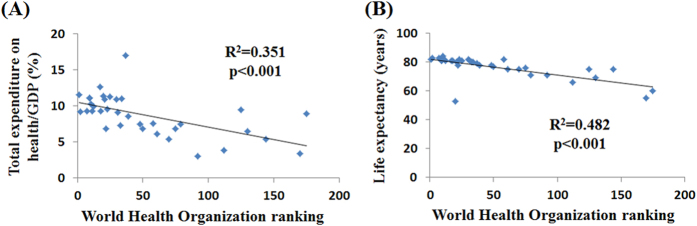
The associations of the World Health Organization ranking with (**A**) the total expenditure on health/GDP and (**B**) life expectancy among 35 countries included in the analysis of prostate cancer.

To further compare the differences in epidemiology among countries, we included the countries with more than 5,000 incident cases (Table 2). The information from the WHO rankings, total expenditures on e/GDP, and life expectancies are summarized in Table 2. As expected, the WHO rankings were significantly associated with both the e/GDP and life expectancy (*R*^2^ = 0.351, *p* < 0.001; *R*^2^ = 0.482, *p* < 0.001, respectively, Fig. 1). Among the 35 countries, the country with the highest WHO ranking was France. For the e/GDP, the highest was the United States (17.0%), and the lowest was Indonesia (3.0%). Among all of the countries, the highest crude rate of incidence was in Sweden (244.9), and the highest crude rate of mortality was in Cuba (54.4).

**Figure 2:**
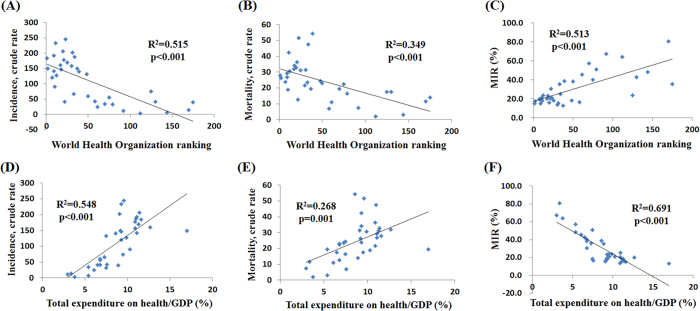
Countries with good World Health Organization rankings have high crude rates of (**A**) incidence and (**B**) mortality of prostate cancer. Additionally, in those with high total expenditures on health/GDP, the crude rates of (**D**) incidence and (**E**) mortality were higher. Higher World Health Organization rankings and total expenditures on health/GDP are associated with favorable MIRs (**C**) and (**F**).

We further correlated the WHO rankings and the e/GDP with the crude rates and MIR for prostate cancer according to country. Countries with better WHO rankings had higher crude rates of incidence and mortality (*R*^2^ = 0.515, *p* < 0.001; *R*^2^ = 0.349, *p* < 0.001, respectively, Fig. 2A and B). The same phenomenon can be found for the correlations of total expenditure on e/GDP with crude rates of incidence and mortality (*R*^2^ = 0.548, *p* < 0.001; *R*^2^ = 0.268, *p* = 0.001, Fig. 2D and E). As for the MIR, the countries with better WHO rankings and e/GDP were associated with favorable MIR (*R*^2^ = 0.513, *p* < 0.001; *R*^2^ = 0.691, *p* < 0.001, respectively, Fig. 2C and F), which means that the WHO ranking and e/GDP were significantly associated with the MIR for prostate cancer.

## Discussion

In this study, we investigated the correlation of MIRs for prostate cancer with cancer care disparities. We used the WHO rankings and e/GDP as indicators of health care disparities among countries. The results suggest that better support of health care expenditures leads to lower MIRs for prostate cancer (Fig. 2). The MIR was calculated with the incidence and mortality rates. This means that besides the dietary, genetic, and environmental contributions of prostate risk, early screenings and advanced surgical and personalized therapy play important roles in improving clinical outcomes which contribute to low MIR^1^,^5^,^14^,^15^,^16^. An observation study found that early detection or a lead time bias of more widespread utilization and earlier introduction of PSA testing in America would cause the differences in incidence and stage distributions over time which would influence the survival^7^. Early detection and appropriate treatments including the advanced surgical intervention equipment and personalized therapies lead to large expenditures in the health care system. This might be the reason that the MIR for prostate cancer is significantly associated with health care disparities between countries. We did not compared the difference of MIR improvement between countries according to the expenditures in the health care system. However, previous studies had shown the advantages of survival rate of developed countries such as America and European countries^7^,^17^. It is no doubt that, in recent years, limited improvement of survival rate in prostate cancer was achieved but they still had better MIRs compared with those countries with low expenditures in the health care system which were shown in our results^7^,^17^.

Regarding the difference in MIRs between regions and countries, a global study has demonstrated that the incidence trend of prostate cancer has increased in both developed regions and developing regions between 1990 and 2013^2^. Our results suggest that prostate cancer has higher incidence and mortality rates in regions with higher development, such as the Americas and Europe. The MIR may be different with an updated database. A previous study has shown that the MIRs for China, Japan, and Korea in 2008 were 0.42, 0.22, and 0.18, respectively, which are similar to our results^18^. Similar trends were also shown in a study from the Asia-Pacific region^9^. These studies indicate that the improvement in the prognosis of prostate cancer was not obvious during these years. However, the high MIR of the African region can be explained by lack of screen, up-staging of disease at the time of diagnosis, the socioeconomic factors, care delivery, and treatment selection that have an African origin^8^. All of these data support our findings that MIR has a role in prediction of care disparities.

The limitations of our study are that we excluded countries with an incidence less than 5,000 cases per year. Additionally, no further clinical information including stage and PSA screening was analyzed. Furthermore, the use of the WHO rankings and e/GDP to represent the health care disparities of countries is not specific; other factors, such as the national health care systems, disparity in access to cancer care, and insurance coverage, should be analyzed. In this study, crude rate was analyzed. We also investigate the WHO rankings and the e/GDP with the age-standardized rate (ASR) for prostate cancer according to country. Countries with better WHO rankings had higher ASR of incidence and mortality (*R*^2^ = 0.328, *p* < 0.001; *R*^2^ = 0.054, *p* = 0.179, respectively). The correlations of total expenditure on e/GDP with ASR of incidence and mortality showed significance in ASR of incidence (*R*^2^ = 0.743, *p* < 0.001; *R*^2^ = 0.068, *p* = 0.697, respectively). The reason of lack significant association between ASR and WHO ranking or e/GDP needs further investigation.

In this study, we demonstrated that the MIR for prostate cancer is associated with health care disparities. Further investigations with greater detail and focus on the data are needed to support our findings. Moreover, future follow-up studies of the MIRs would be helpful in monitoring improvements in prostate cancer care among countries.

## Additional Information

**How to cite this article**: Chen, S.-L. *et al*. Prostate Cancer Mortality-To-Incidence Ratios Are Associated with Cancer Care Disparities in 35 Countries. *Sci. Rep.* **7**, 40003; doi: 10.1038/srep40003 (2017).

**Publisher's note:** Springer Nature remains neutral with regard to jurisdictional claims in published maps and institutional affiliations.

## Figures and Tables

**Table 1:** Summary of prostate cancer crude rates of incidence, mortality, and mortality-to-incidence ratios according to region.

Region	Prostate cancer				
	Incidence		Mortality		MIR(%)
	Number	Crude rate	Number	Crude rate	
World	1094916	30.8	307481	8.6	28.1
Development					
More developed regions	741966	122.4	142014	23.4	19.1
Less developed regions	352950	12.0	165467	5.6	46.9
Development categories					
Very high human development	734128	129.0	131685	23.1	17.9
High human development	195839	38.2	72623	14.2	37.1
Medium human development	115942	6.4	63739	3.5	55.0
Low human development	47809	7.3	39096	6.0	81.8
WHO region categories					
WHO Africa region	51689	11.8	37486	8.5	72.5
WHO Americas region	412739	87.6	85425	18.1	20.7
WHO East Mediterranean region	18585	5.8	12141	3.8	65.3
WHO Europe region	419915	96.1	101419	23.2	24.2
WHO South-East Asia region	38515	4.1	24932	2.6	64.7
WHO Western Pacific region	153167	16.2	45977	4.9	30.0
Continent					
Africa	59493	11.1	42802	8.0	71.9
Latin America and Caribbean	152403	51.2	51313	17.2	33.7
North America	260336	150.2	34112	19.7	13.1
Asia	196190	9.0	82676	3.8	42.1
Europe	400364	112.0	92328	25.8	23.1
Oceania	26130	138.3	4250	22.5	16.3

**Table 2:** Summary of the World Health Organization rankings, total expenditures on health/GDP, life expectancy, and incidence/mortality in crude rates and case numbers of countries with prostate cancer incidences of more than 5,000 cases per year.

Country	Ranking	Total expenditure on health/GDP (%)	Life expectancy	Incidence		Mortality		MIR(%)
				Number	Crude rate	Number	Crude rate	
Indonesia	92	3.0	71	13663	11.2	9191	7.5	67.3
Nigeria	170	3.4	55	11944	14.2	9628	11.4	80.6
Mexico	61	6.1	75	14016	24.5	6367	11.1	45.4
South African	175	8.9	60	9957	39.6	3539	14.1	35.5
Russian	130	6.5	69	26885	40.7	11480	17.4	42.7
Turkey	70	5.4	75	12650	34.0	7231	19.5	57.2
India	112	3.8	66	19095	2.9	12231	1.9	64.1
China	144	5.4	75	46745	6.6	22603	3.2	48.4
Poland	50	6.8	77	11029	59.7	4242	23.0	38.5
Portugal	12	9.9	81	6622	127.7	1582	30.5	23.9
Colombia	22	6.8	78	9564	40.9	2934	12.5	30.7
Chile	33	7.3	80	5681	66.0	2029	23.6	35.7
Cuba	39	8.6	78	7931	140.2	3080	54.4	38.8
Argentina	75	6.8	76	11202	55.7	4489	22.3	40.1
Brazil	125	9.5	75	72536	74.4	17218	17.6	23.7
Germany	25	11.3	81	68262	169.7	12548	31.2	18.4
France	1	11.6	82	56841	184.0	8606	27.9	15.1
Italy	2	9.2	83	44525	149.0	7814	26.2	17.5
Spain	7	9.3	83	27853	120.5	5481	23.7	19.7
Switzerland	20	11.4	53	7851	206.3	1248	32.8	15.9
Japan	10	10.3	84	55970	90.9	11644	18.9	20.8
Netherlands	17	12.7	81	13300	160.2	2650	31.9	19.9
Sweden	23	9.6	82	11596	244.9	2444	51.6	21.1
South Korea	58	7.6	82	10351	42.7	1696	7.0	16.4
Belgium	21	10.9	80	9393	177.6	1913	36.2	20.4
Czech	48	7.5	78	6848	132.0	1268	24.4	18.5
Ukraine	79	7.5	71	6637	32.1	3374	16.3	50.8
Austria	9	11.1	81	5833	141.6	1105	26.8	18.9
Norway	11	9.3	82	5789	232.9	1054	42.4	18.2
Denmark	34	11.0	80	5205	187.6	1316	47.4	25.3
USA	37	17.0	79	233159	149.5	30383	19.5	13.0
United Kingdom	18	9.3	81	45406	146.7	10595	34.2	23.3
Canada	30	10.9	82	27087	157.4	3722	21.6	13.7
Austria	9	11.1	81	21966	192.2	3333	29.2	15.2
Finland	31	9.1	81	5366	202.2	832	31.4	15.5
